# Characterizing the conformational landscape of MDM2-binding p53 peptides using Molecular Dynamics simulations

**DOI:** 10.1038/s41598-017-15930-4

**Published:** 2017-11-15

**Authors:** Shilpa Yadahalli, Jianguo Li, David P. Lane, Shachi Gosavi, Chandra S. Verma

**Affiliations:** 1 Simons Centre for the Study of Living Machines, National Centre for Biological Sciences, Tata Institute of Fundamental Research, Bellary Road, Bangalore, 560065 India; 2 Bioinformatics Institute, A*STAR (Agency for Science, Technology and Research), 30 Biopolis Street, #07-01 Matrix, Singapore, 138671 Singapore; 3 Manipal University, Madhav Nagar, Manipal, 576104 India; 4 p53 Laboratory, A*STAR (Agency for Science, Technology and Research), 8A Biomedical Grove, #06-04/05 Neuros/Immunos, Singapore, 138648 Singapore; 5 Singapore Eye Research Institute, 11 Third Hospital Avenue, #06-00, Singapore, 168751 Singapore; 6 Department of Biological Sciences, National University of Singapore, 16 Science Drive 4, Singapore, 11758 Singapore; 7 School of Biological Sciences, Nanyang Technological University, 60 Nanyang Drive, Singapore, 637551 Singapore

## Abstract

The conformational landscapes of p53 peptide variants and phage derived peptide (12/1) variants, all known to bind to MDM2, are studied using hamiltonian replica exchange molecular dynamics simulations. Complementing earlier observations, the current study suggests that the p53 peptides largely follow the ‘conformational selection’ paradigm in their recognition of and complexation by MDM2 while the 12/1 peptides likely undergo some element of conformational selection but are mostly driven by ‘binding induced folding’. This hypothesis is further supported by pulling simulations that pull the peptides away from their bound states with MDM2. This data extends the earlier mechanisms proposed to rationalize the entropically driven binding of the p53 set and the enthalpically driven binding of the 12/1 set. Using our hypothesis, we suggest mutations to the 12/1 peptide that increase its helicity in simulations and may, in turn, shift the binding towards conformational selection. In summary, understanding the conformational landscapes of the MDM2-binding peptides may suggest new peptide designs with bespoke binding mechanisms.

## Introduction

**Figure 1:**
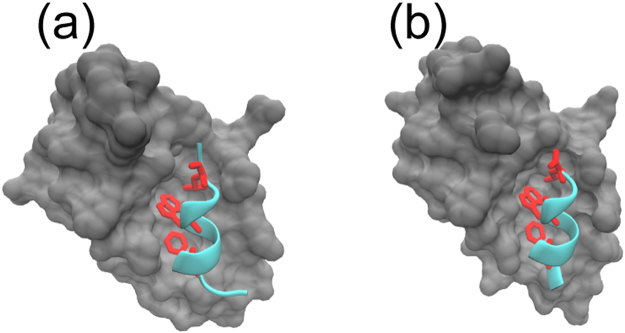
The crystal structures^8,13^ of (**a**) the p53 wild type and (**b**) the 12/1 peptide in complex with the target protein MDM2. MDM2 is shown as a grey surface and the peptides are shown in cyan cartoon; key residues critical for binding to MDM2 (F19, W23, L26 for p53 WT; F4, W7, L11 for 12/1) are shown as red sticks. The structure for (**a**) was taken from the PDB ID 1YCR while for (**b**) was taken from PDB ID 1T4F. Structures are drawn using VMD^58^.

p53 is a transcription factor that plays a central role in cell cycle regulation. The levels of p53 are maintained mostly by the E3 ubiquitin ligase murine double minute (MDM2) protein which targets p53 for proteasomal degradation. The N-terminal domain of MDM2 interacts with the N-terminal transactivation domain (NTD) of p53 resulting in an allosteric switch that results in the ubiquitination and subsequent degradation of p53^1^. In normal cells, stress signals abrogate the interactions between the two N-terminal domains through post-translational modifications, resulting in the activation of p53. However, over-expression of MDM2, characteristic of several human tumors, results in the suppression of p53 and the loss of its function^2^. The discovery that MDM2-interacting mimics of the p53 NTD, including small molecules and peptides, can disrupt the MDM2-p53 interaction and reactivate p53, is being pursued as a therapeutic approach in oncology^3,4^. The advantage of peptidic inhibitors is that they can easily be fine-tuned for better affinity and specificity and also appear to tackle the emergence of resistance^5^. Both small molecules and peptides mimic a crucial recognition element of the NTD of p53. The NTD of p53 (residue numbers 1 to 58), which is an intrinsically disordered region, is thought to exist in equilibrium between disordered and partially helical conformations^6,7^. The segment Q_16_ETFSDLWKLLP_27_ of NTD, referred to as the ‘p53-peptide’, forms a stable amphipathic α-helix upon binding to a hydrophobic cleft of MDM2^8^ (Fig. 1) with Phe19, Trp23 and Leu26 serving as essential recognition elements lying on one face of the amphipathic helix and embedding into the hydrophobic groove of MDM2. All small molecule and peptidic inhibitors to date have been found to mimic the interactions between these 3 NTD residues and MDM2. The ‘p53-peptide’ has been shown to be mostly disordered with a small segment adopting helical structures in solution^9^. Variants of this peptide have been generated using empirical designs, computational designs, phage display, mRNA display etc. and have been shown to compete out p53 binding to MDM2 with up to 200-fold increases in affinity^10,11^. In one of the earliest phage display experiments, a high affinity peptide, referred to as 12/1 (M_1_PRFMDYWEGLN_12_)^12^ was found with the 3 essential MDM2-binding NTD residues numbered Phe4, Trp8, Leu11. The p53-peptide and the 12/1 peptide both adopt similar helical conformations upon binding to MDM2, as observed from their crystal structures (Fig. 1 PDB IDs: 1YCR^8^, 1T4F^13^). The observation that the p53 peptide has a Pro while the 12/1 peptide has an Asn residue at its C-terminus lead to an investigation of the importance of this position^14^. A combination of experiments and simulations were carried out to study mutants at this position. The study revealed that while the two classes of peptides bind to MDM2 with similar affinities, the binding free energies of the p53 peptides mostly have large favorable entropic contributions while that of the 12/1 set have large enthalpic contributions. The p53 peptides were shown by CD spectroscopy to have higher helicity compared to the 12/1 peptides in their unbound forms. Experiments elsewhere had demonstrated that the Pro to Ser mutation in p53 resulted in increased helicity and higher affinity^15^. This led to the hypothesis that the pre-organization of the p53 peptides into helical conformations results in smaller entropies of reorganization (and hence reduced entropic penalties) associated with the free energies of binding to MDM2. In contrast, the 12/1 set is more disordered in solution, incurs a large reorganizational penalty upon binding, and compensates by making more stabilizing interactions with MDM2 (thus contributing favorably to the free energy of binding). If this hypothesis is true, it also raises the possibility of modulating the sequence of the peptides to generate higher affinity either by improving the enthalpic interactions of the p53 set or increasing the helicity and thereby reducing the reorganizational penalty of the 12/1 set. Here, we provide further support for this hypothesis, by an exhaustive characterization of the conformational landscapes of the p53 and 12/1 peptides in their ‘unbound’ form (uncomplexed) using replica exchange molecular dynamics simulations (the sequences of the peptides and the nomenclature used in this study is shown in Table 1). We then use an understanding of these conformational landscapes to generate a variant of the 12/1 peptide which has a higher helicity in simulations than the original 12/1 peptide while maintaining a similar number of stabilizing interactions. Such variants combine the stabilizing characteristics of both the p53 and the 12/1 peptides and may thus have a higher affinity for MDM2.

**Table 1:** Peptides studied in the current work. F19, W23, L26 (1YCR:B: PDB numbering) are known to be important for interaction with MDM2 and are conserved in all the peptide sequences.

Peptide sequence	Name	K_d_ values in nM	ΔG (kcal/mol)
Q_16_ETFSDLWKLL**P**_**27**_	p53-wild type (WT)	1543.2 ± 90	−7.79 ± 0.03
QETFSDLWKLL**S**	p53-P27S	75.76 ± 10.45	−9.54 ± 0.08
QETFSDLWKLL**N**	p53-P27N	168 ± 9.24	− 9.076 ± 0.27
MPRFMDYWEGL**N**	12/1	239.8 ± 53.7	−8.87 ± 0.12
MPRFMDYWEGL**S**	12/1-N12S	18.83 ± 5.03	−10.35 ± 0.14

K_d_ is the dissociation constant of the interactions between MDM2 and the peptides. ΔG is the binding free energy. Values of K_d_ and ΔG are taken from the ITC data from a previous study^14^.

## Methods

### Hamiltonian Replica Exchange Method

For the simulations, the unfolded models (random coil conformations) of the peptides were generated (see next subsection for details) and were subjected to replica exchange molecular dynamics (REMD), which have been shown to efficiently enhance sampling of the conformational space of proteins^16^. In replica exchange simulations, multiple replicas differing in temperature or hamiltonians are run in parallel and adjacent replicas are exchanged at regular intervals based on the Metropolis criterion, ensuring a Boltzmann distribution of the resultant conformational ensemble. In this study, the replicas differ in the hamiltonian whereby the solute-solute and solute-solvent interactions are perturbed. Hence, this method is also known as replica exchange by solute tempering^16^ and is employed because we are mainly interested in the solute conformation. This method is computationally less expensive than temperature based replica exchange^17^. We followed the implementation of hamiltonian replica exchange in GROMACS as described by others^17^. The rescaling of solute-solute and solute-solvent interactions is achieved by a coupling parameter λ. To choose the appropriate λ, we carried out conventional Molecular Dynamics (MD) simulations at λ = 0.4, 0.5, 0.7 and found that for λ $\ge$ = 0.5, the peptide unfolded within 20 ns. Hence, eight replicas with equally spaced λ values between 0 and 0.5 were chosen. This results in sufficient overlap in the potential energy distributions of adjacent replicas and an acceptance ratio in the range of 15–22%. The peptide conformations at the two extreme λ values (0 and 0.5) are shown in Fig. S1. The replica at λ = 0 corresponds to the real/unperturbed hamiltonian equivalent to conventional MD simulations and rest of the replicas are introduced for enhancement of solute conformational sampling. We ran each replica for 300 ns and found that the simulations had converged within this time frame. Convergence was tested by examining distributions of Root Mean Square Deviation (RMSD), Radius of gyration (Rg), and the associated Free Energy Surfaces (FES) (Fig. S2). It should be highlighted that the analyses of these simulations are always carried out for the conformations sampled at λ = 0 (unperturbed Hamiltonian).

### Simulation details

All simulations were performed with the GROMACS 4.5^18^ MD suite. The starting structure for each simulation was an unfolded random conformation of the peptide being simulated, and was generated by running MD simulations at a higher temperature (350 K for 50 ns). We used the AMBER99SB force field with TIP3P water model^19^. One Na^+^ ion was added to each system to neutralize the net charge. The Verlet leapfrog algorithm was used to propagate the dynamics of the system at a timestep of 2 fs. Bond lengths between heavy atoms and hydrogen atoms were constrained with the LINCS algorithm^20^. The Particle Mesh Ewald method^21^ was applied to simulate the long-range electrostatic interactions. A modified Berendsen thermostat (V-rescale) was used to maintain the temperature (300 K) and Parrinello-Rahman pressure coupling was used to maintain the pressure (1 bar). Initial velocities were generated according to the Maxwell distribution. Periodic boundaries were used in all directions. The cutoff distances for the short-range neighbor list and van der Waals (vdW) interactions were 1 nm and 1 nm respectively. Prior to the production runs, each replica was equilibrated for 200 ps in the NVT and for 500 ps in the NPT ensembles. Coordinates between replicas were exchanged every 500 steps (10 ps) and the production run was carried out for 300 ns at 300 K in the NPT ensemble.

### Center of Mass (COM) pulling

The peptide is pulled at a constant velocity (0.1 nm per ns) with a force applied to its Centre of Mass (COM) in a direction away from the binding site, perpendicular to the peptide-MDM2 interacting surface. To reduce pulling induced conformational perturbations of MDM2, positional restraints were applied to all backbone atoms beyond 8 Å from the binding pocket; this maintains the overall conformation of MDM2 while the residues in the binding pocket are allowed to be flexible. We carried out 10 replicate simulations for each of the two complexes: MDM2:p53-P27S and MDM2:12/1. These complexes were modeled from the crystal structure 1YCR where p53 peptide sequence was mutated in Pymol (Version 1.4.1 Schrödinger, LLC) to get the new sequence. We used the 1YCR structure rather than the 1T4F structure for the 12/1 peptide to ensure that any bias arising from the conformational differences between two different crystal structures was minimized. The structure of MDM2 in 1YCR covers the sequence from residues 25 to 109 (the exclusion of the region 1–24 is explored in Discussion).

### Secondary structural analysis

The overall secondary structural content in our simulations was estimated by using DSSP^22,23^ which is implemented in the ‘do_dssp’ plugin available in GROMACS. The helical population accessed in the simulations was computed using the following metrics:

i) The fraction of residues adopting α-helical conformation over each simulation (Table 2).

**Table 2:** Percentage of secondary structure population in all the peptides from their replica exchange simulations.

Peptide	α helix	3_10_ helix	β-sheet
p53-WT	17	9	0
p53-P27S	26	7	1
p53-P27N	32	7	0
12/1	9	6	3
12/1 N12S	7	3	7
ii) The fraction of snapshots from each simulation when a peptide is at least 70% helical for a given snapshot (Table 3).

**Table 3:** Quantifying the populations of helical structures in simulations of all the peptides from replica exchange simulations. The values shown are percentage of snapshots in replica exchange simulations of the peptides (population).

Peptide	RMSD < 2 Å w.r.t. ideal helix	helicity more than 70% per structure
p53- WT	7.7	1.64
p53-P27S	18.2	6.5
p53-P27N	17.4	6.7
12/1	2.1	0.24
12/1 N12S	1.1	0.09
iii) The fraction of each simulation when the peptide was within 2 Å RMSD of an ideal helix (defined as φ: −57° +/−7°, ψ: −47°+/−7°) (Table 3).

All data analyzed during this study are included in this published article (and its Supplementary Information).

### 2D Free energy surfaces (2DFESs)

2DFESs were calculated as follows. For each snapshot from our simulations, two parameters were calculated: Root Mean Square Deviation (RMSD) of the conformation in that snapshot relative to the conformation of the peptide when bound to MDM2 (at the Cα atom level) and the Radius of gyration (Rg) of that snapshot. These were then binned to get 2D histograms. The 2DFES was generated by calculating the negative logarithms of these 2D-histogram values (-ln(populations)). The free energy of the basins on the 2DFESs are referred to as clusters here. We also plot 2DFES using other parameters such as (φ, ψ) dihedral angles in the peptides.

## Results

### p53 peptides are more helical than the 12/1 peptides

The peptides investigated in this study bind to MDM2 in similar helical conformations and with similar binding free energies. However, the p53 peptides and the 12/1 peptides have different enthalpic and entropic contributions to these binding free energies^14^. In order to understand the conformational landscapes of the peptides and hence the contribution of peptide conformational dynamics to the free energy of binding, we performed REMD simulations of the peptides in their unbound forms. We then analyzed the structural content in the peptide trajectories using both global and residue-level metrics to understand the similarity of the unbound conformations to the MDM2-bound helical conformations.

**Figure 2:**
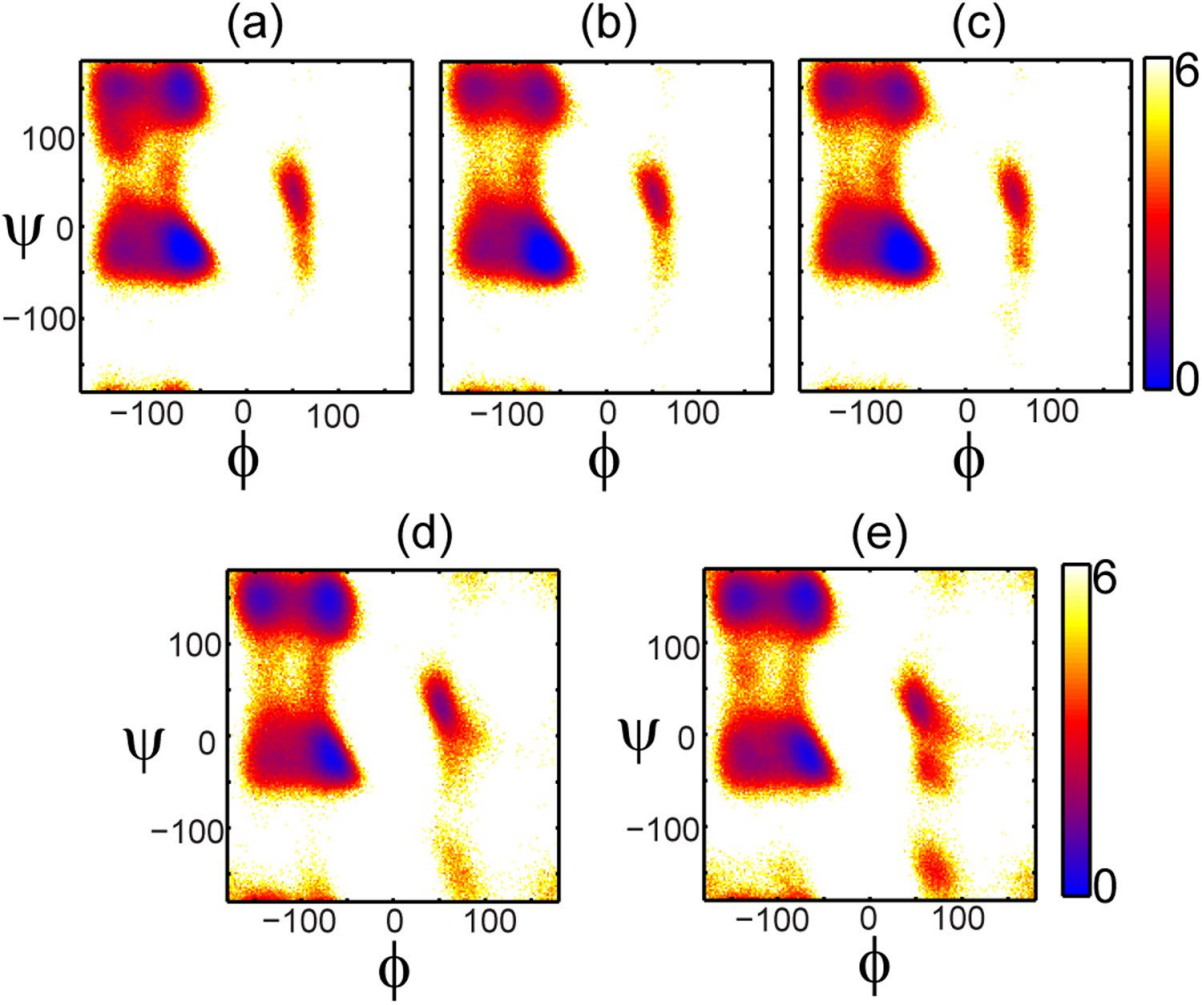
Ramachandran 2DFES plots of the peptides. The distribution of the conformations of the peptides (-ln(population)) from the replica exchange simulations plotted as a function of the φ (X-axis; in degrees) and ψ (Y-axis; in degrees) diherdal angles for all the peptides. The distributions are colored from blue (highest density of structures) through red to yellow (lowest density of structures); color scheme used is shown by the color bars on the right. (**a**) p53-WT (**b**) p53-P27S (**c**) p53-P27N (**d**) 12-1 (**e**) 12-1 N12S. The α-helical population ((φ,ψ) ~(−60°, −45°)) is higher in the p53 peptides. 12/1 peptides have similar α-helical and β-sheet ((φ, ψ) ~(−135°, 135°)) populations. 12/1 peptides also show larger populations of PPII helices ((φ, ψ) ~(−75°, 150°)).

DSSP calculations analyzed using three different ways (fraction of residues in an α-helical conformation, Table 2; fraction of trajectory where at least 70% of the peptide is helical, Table 3; and fraction of trajectory where the peptide is structurally similar to an ideal helix, Table 3), show that the p53 peptides have a greater helical content than the 12/1 peptides, which also adopted other structured states. The observation that p53-27S is more helical than p53-WT and other results are largely in agreement with published CD data^14^. We also plotted 2DFESs of the φ, ψ dihedral angles (Ramachandran plots), for each peptide. α-helical structures in such plots are located at (φ, ψ) of (−60°,−45°), β sheets at (φ, ψ) of (−135°, 135°), PPII structures at (φ, ψ) of (−75°, 150°) and left handed α-helical structures at (φ, ψ) of (+60°, +45°). From Fig. 2, it is observed that the p53-WT peptide adopts α-helical and PPII conformations while the p53-mutant peptides adopt largely α-helical conformations. In contrast, the 12/1 peptides populate α-helical, β-sheet and PPII conformations, without any preference for a particular secondary structure.

**Figure 3:**
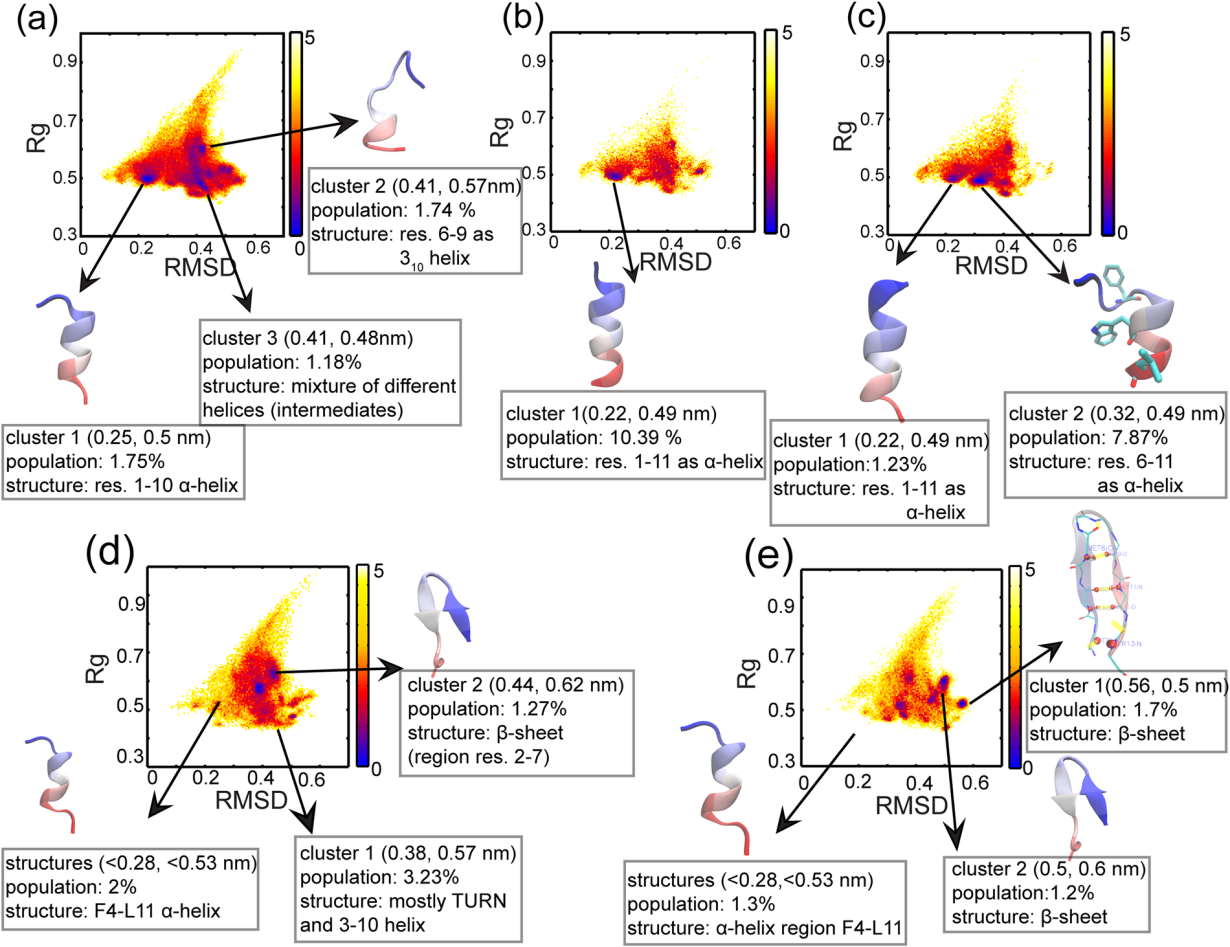
Conformational landscapes of the peptides. The 2D free energy surfaces (2DFES) show the distributions of various conformations (-ln(population)) colored from blue (highest density of structures) to red to yellow (lowest density of structures); the color scheme used is shown by the color bars on the right. The X-axes represent the root mean square deviation (RMSD; in nm) from the bound form of the p53-WT peptide crystallized in a complex with MDM2 (1YCR: chain-B) calculated over the Cα atoms. The Y-axes represent the radius of gyration (Rg; in nm) of the peptides. Representative structures from significantly populated clusters are shown with arrows indicating their positions on the 2DFES. The structures are colored as blue to red from the N to the C-termini. Information about the clusters including the average (RMSD, Rg) coordinates of the ‘cluster centre’ and their populations are mentioned below each structure. (**a**) p53-WT (**b**) p53-P27S (**c**) p53-P27N (**d**) 12-1 (**e**) 12-1 N12S. The structural representative of the intermediate found in p53-P27N has the key binding residues shown as sticks in panel (c); Phe19 is not in a helical conformation in this intermediate ensemble. This intermediate is also found in p53-WT with less than 1% population. The structural representative of the β-hairpin cluster in the 12/1 N12S peptide has its hydrogen bonds shown as yellow dotted lines in panel (e).

The DSSP calculations and Ramachandran plots give the average properties of the peptide ensembles. For a detailed structural characterization we clustered them to identify different sub-populations present in the various sampled ensembles. This was achieved by plotting the 2D free energy surfaces with RMSD relative to the bound form of the peptide and Rg, as the two coordinates (see Methods). Representative structures from significantly populated clusters are shown on the 2DFESs. Information about the clusters including the average (RMSD, Rg) coordinates of the ‘cluster centre’ and their populations are mentioned below each structure (Fig. 3). Here too, Once again it is clear that the p53 peptides have a higher population of helical conformations than the 12/1 peptides. The regions of p53 that are helical are: residues 1–10 in WT and residues 1–11 in P27S and P27N. We also observe one folding intermediate in the P27N simulations. A structural representative of this intermediate is shown in Fig. 3c with the key binding residues drawn as sticks. We see that the Asp21- Leu26 region is helical in this intermediate but Phe19, one of the key binding residues, is not in the helical form (Fig. 3c). This intermediate is also found in p53-WT, albeit with a reduced population.

The 12/1 peptides sample smaller populations of helices compared to the p53 peptides and they also sample shorter helices extending from Phe4 to Gly10. Nevertheless, the three key binding residues Phe, Trp and Leu are part of this helical region. Overall, the 2DFESs of the 12/1 peptides are more rugged compared to the p53 peptides, with populations of various non-helical secondary structures. Interestingly, the 12/1 peptides also sample β-hairpin structures, which are not seen in the p53 peptides; we show a structural representative from the 12/1 N12S peptide simulations in Fig. 3e, since this mutant was characterized by the most stable β-hairpin population. We next examine the secondary structural propensities of individual amino acids in the peptides to help guide the engineering of specific conformational properties.

### Effects of single amino acid changes on the peptide conformations

**Figure 4:**
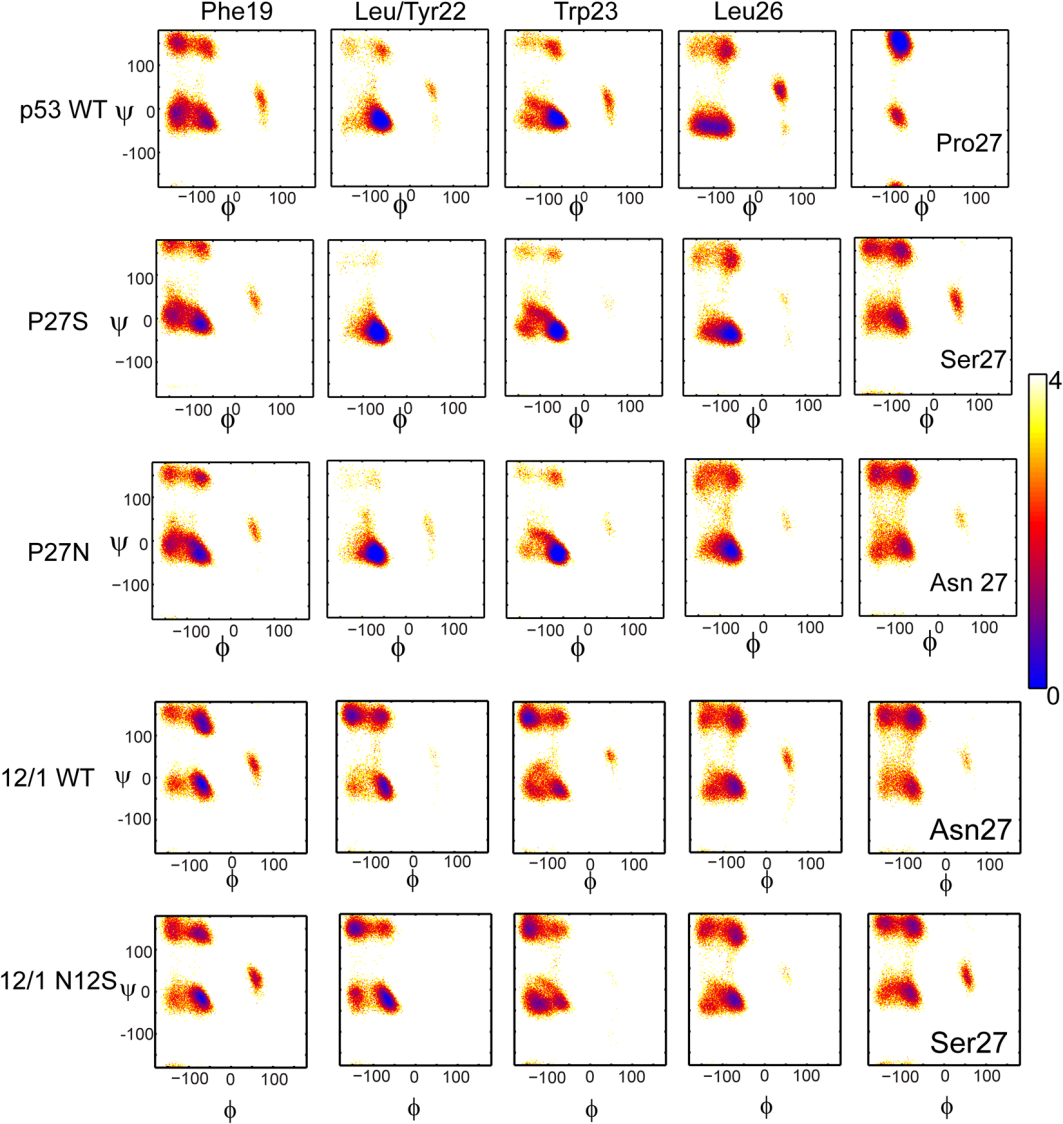
Ramachandran 2DFES plots for the key residues from the p53 peptides. The distribution of the conformations (-ln(population)) of the key binding residues from the p53 peptides from the replica exchange simulations plotted as a function of the φ (X-axis; in degrees) and ψ (Y-axis; in degrees) dihedral angles. The distributions are colored from blue (highest density of structures) through red to yellow (lowest density of structures); color scheme used is shown by the color bars on the right. The rows represent a peptide and columns represent the key residues. The last column is from residue 27 in the peptides which is Pro in WT, Ser in P27S and Asn in P27N, Asn in 12/1 peptide, and Ser in 12/1 N12S. The α-helical structures have (φ, ψ) of (−60°, −45°); β sheet has (φ, ψ) of (−135°, 135°) and PPII structures have (φ, ψ) of (−75°, 150°). Left handed α-helical structures ((φ, ψ) ~ (+60°, +45°)) are also marked. As expected, Pro27 from p53-WT is present mainly in a PPII conformation. Mutating this Pro to Ser and Asn increases the helicity of both the 26th and the 27th residues. Although Phe19, Leu22 and Trp23 are mainly α-helical in the p53 peptides, Leu22 is the most constrained residue in this region. Phe4 in 12/1 (corresponding to Phe19 in p53) occupies both α-helical and the PPII regions but in the N12S mutant the α-helical population has increased. Tyr7 (Leu22 in p53) populates both β-sheet and α-helix. Trp8 (Trp23 in p53) populates mainly β sheet conformations in the 12/1 peptides. Leu11 (Leu26 in p53) has a higher population of α-helix as compared to the other residues in 12/1.

To examine the conformations adopted by individual amino acids, Ramachandran maps of the 3 key binding residues (Phe19, Trp23, Leu26), Leu/Tyr22 and the C-terminal residue were plotted for each peptide (Fig. 4). The effects of the single residue changes at the C-termini of the peptides are clearly visible. Pro27 (with its pyrrolidine ring) reduces the conformational freedom of the preceding Leu26 and both occupy restricted conformations in the Ramachandran plot. Further, Proline cannot be a hydrogen bond donor and acts as a helix disruptor. Thus, mutating Proline to any other residue is expected to increase helicity. This is clearly seen for the 3 key residues (Phe19, Trp23, Leu26) which all adopt more helical conformations for both P27S and P27N mutants (Fig. 4). In addition to the 3 key residues, we also observe that Leu22 adopts helical conformations; indeed it is the most helical of the residues. To analyze this further, we calculated the probability of hydrogen bond (h-bonds) formation in the p53-peptide simulations using the ‘g_hbond’ utility of GROMACS with a cutoff of 2.8 Å. For P27S (Fig. S3), we find that 5 out of the top 10 occurrences of h-bonds correspond to those formed by Leu22.

Ramachandran plots for the key residues of the 12/1 peptides show that Phe4 in 12/1 (structurally homologous to Phe19 in the p53 peptides) occupies both α-helical and PPII regions while in 12/1 N12S, the α-helical population is increased. Trp8 (Trp23 in p53 peptides) populates mainly β-sheets, Leu11 (Leu26 in p53 peptides) is mostly in helical conformations while Tyr7 (Leu22 in p53) populates both β-sheet and α-helical conformations. In summary, the residues from p53 peptides prefer to be in α-helical conformations while residues in the 12/1 peptides adopt multiple conformations.

### p53-peptides retain helicity when “pulled” from their binding sites

**Figure 5:**
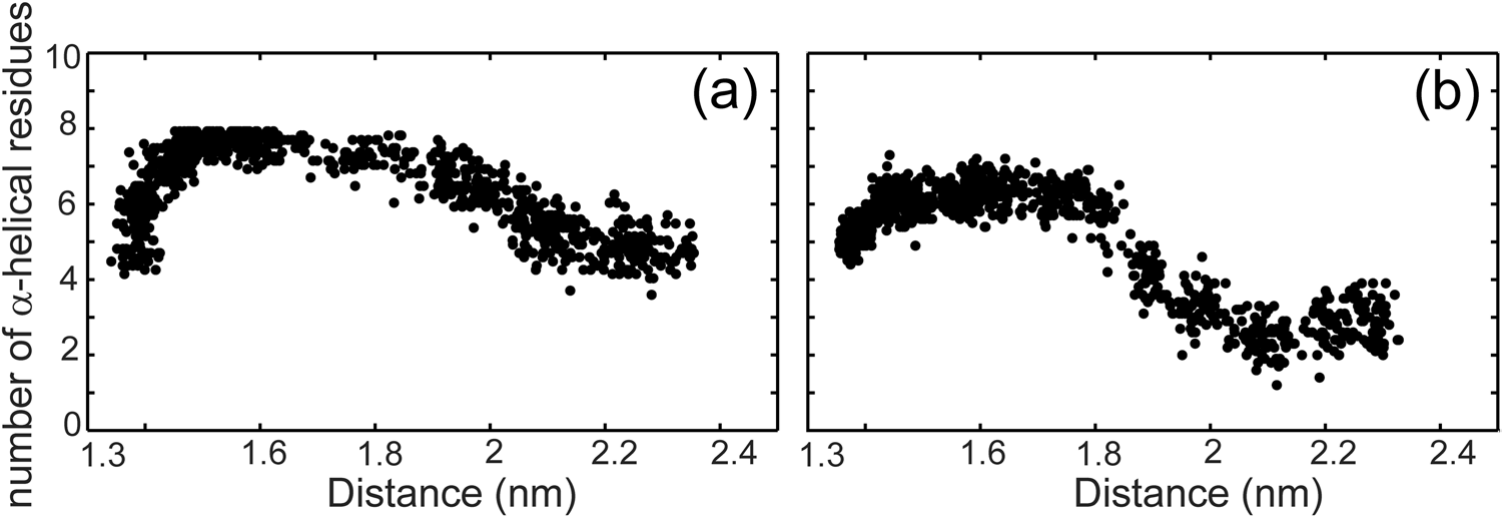
The behavior of the secondary structures of the peptides as they are pulled away from the MDM2 binding site. Here we show the average behavior of the peptides across ten replicates of the pulling simulations for (**a**) p53-P27S and (**b**) 12/1. X-axis is the distance between the centers of masses of the peptide atoms and the MDM2 atoms. Y-axis is the number of residues in an α-helical conformation as calculated by DSSP. These values are calculated for each frame of a trajectory and are averaged across all 10 trajectories for a given simulation time point. Starting distance between the centers of masses of the peptide and MDM2 is 1.33 nm. p53-P27S retains at least 50% helicity at longer distances while the 12/1 peptide loses helicity in most of the replicates.

To examine how the peptides behave away from their MDM2 binding sites, we also carried out pulling simulations of the peptides from their complexed states (with MDM2). We chose to focus on p53-P27S and 12/1 because these two have similar binding affinities to MDM2 and the CD data unambiguously suggests that p53-P27S is helical while 12/1 is not, thus enabling a comparison between the two extreme conformations adopted by these peptides. If the peptide adopts structures close to the bound form (here, helical) away from the binding site, then recognition and complexation is hypothesized to be predominantly driven by conformational selection. If however, the conformation of the peptide away from the binding site departs from the helical structure then the process of binding is driven largely by binding induced folding^24^. The average number of residues in an α-helical conformation (calculated by DSSP) as a function of the distance between the centers of masses of the peptides and the MDM2 atoms is shown in Fig. 5. These values are calculated for each frame of a trajectory in which the peptides are pulled away from their bound states and are averaged across all 10 replicate trajectories for a given simulation time point. The starting distance between the centers of masses of the peptide and MDM2 is ~1.33 nm. It is apparent from Fig. 5 that P27S maintains helicity at longer distances than 12/1. In Fig. S4 we show the behavior of the 10 individual replicates for both peptides. The P27S peptide retains its helical conformation in 7/10 replicates. The 12/1 peptides remains helical only for 5 ns, at a distance of about 1.65 nm away from the MDM2 binding surface. We have also carried out error estimates of our pulling simulation data using the jackknife resampling^25^ method (Fig. S8). It is clear that the error bars (in panels a, b, c of Fig. S8) increase as the peptides are pulled away from MDM2, reflecting the increasing fluctuations/disorder in the peptide conformations.

In summary, the MD simulations together support our previously hypothesized binding mechanism: the p53 peptides retain their helicity in the unbound form and are thus likely to follow the ‘conformational selection’ paradigm in their recognition and complexation of MDM2, while the more conformationally labile 12/1 peptides possibly undergo some element of conformational selection but are mostly driven by ‘binding induced folding’.

## Discussion

### p53 and 12/1 peptides bind by different mechanisms to their target protein MDM2

The conformational propensities of peptides are known to influence the mechanisms of folding and binding^26–28^. Peptides are thought to largely exist as heterogeneous ensembles of interconverting conformations, thus making detailed experimental characterization a challenge. Spectroscopic techniques like CD are useful in describing secondary structures but only provide ensemble averages. NMR studies can provide high-resolution structural information, but are limited in scope, especially for highly disordered systems^29^. Atomistic modeling and simulations have become useful tools that complement experimental techniques in providing exquisite details in space and time^30^.

A set of peptides derived from the protein p53 was studied for their ability to interact with the protein MDM2^13^. The peptides were classified into two sets: the set referred to as the p53 set are single point mutants of the WT p53 sequence; the set referred to as 12/1 are single point mutants of a phage-display variant of p53, called 12/1. Both sets of peptides, p53 and 12/1, adopt very similar conformations when bound to MDM2 (as seen in the crystal structures, PDB IDs 1YCR^8^ and 1T4F^13^ Fig. 1), and also bind to MDM2 with similar free energies. The free energies were reported to be dominated by the entropic component for the p53 set but enthalpically for the 12/1 set^14^. This was attributed to the variations in the helical propensities of the peptides. From this data, it was reasonable to hypothesize that both sets of peptides bind to MDM2 by different mechanisms giving rise to similar affinities^31^. We explore this further by studying the conformational landscapes of the peptides in solution (in their unbound form), by carrying out an exhaustive simulation study.

REMD simulations show that both sets of peptides adopt partially folded helical states. While helicity dominates the landscape of the p53 set, the 12/1 set of peptides additionally adopt a variety of conformations including several non-helical states. This is in agreement with the conclusions of the previous study^13^ which speculated that sequestration by MDM2 of the helically pre-organized p53 set requires lower entropic penalties compared to the largely non-helical 12/1 peptides. The picture that emerges is one of ‘conformational selection’ as the dominant mechanism of binding of the p53 set with ‘binding induced folding’ characterizing the binding of the 12/1 peptides. This is consistent with an earlier study that had combined spectroscopy with MD simulations to examine the P27S mutation of the WT p53. They found that the peptides adopted helical conformations in solution and speculated that binding of the p53-peptides to MDM2 may involve “conformational selection^32^.

It has been suggested that disordered segments, characterized by larger radii of gyration, have a kinetic advantage in inter-molecular associations by providing a larger capture radius (conformations conducive to binding)^33,34^ (aka ‘fly casting’). It is clear that the distribution of Rg is wider for the 12/1 set compared to the p53 set (Fig. S5). Additionally, 12/1 has a higher Polyproline helix II (PPII) content. PPII is a left-handed helix defined by the (φ, ψ) dihedral angle cluster with the distribution maximum at (−75°, 145°) and are found extensively distributed in disordered proteins/peptides^35,36^. These helices do not have regular patterns of intra-chain hydrogen bonds, adopting structures that are more extended than in helices, sheets etc. (3 residues per turn and rise per residue is 3.1 Å; whereas α-helix has 3.6 residues per turn and rise per residue is 1.5 Å in the α-helix). Our simulations show that 12/1 has a higher tendency to populate the PPII helix than p53-WT (Figs 2 and 4). It is likely that the higher affinity of the 12/1 peptides for MDM2 may originate partly in the kinetic advantage resulting from their extended states. These conclusions are further strengthened by results from our pulling simulations which show that the p53-P27S peptide retains helicity whereas the 12/1 peptide loses helicity as it is pulled away from the binding region (Fig. 5). Together these results suggest that the 12/1 peptides are extended in solution, but are funneled into adopting partial helical structures in the vicinity of MDM2.

At the individual residue level, it is interesting that Leu22 in the p53 peptides is the most constrained in the helical form while a Tyr, adopting a β-sheet conformation, has been selected in 12/1 in the phage selection (Fig. 4). Tyr22 is known to form a higher number of hydrophobic interactions with MDM2 than does Leu22 in p53^37,38^. This increased interaction may compensate for the entropic loss resulting from lower helicity and may account for its selection in the phage display process.

### Simulations predict the presence of an intermediate structure in p53 peptides

The conformational landscapes of the peptides in our REMD simulations are populated by a variety of conformations including different types of helices (such as 3_10_, pi helix). A particular state, where the Asp21 to Leu26 region is helical, is significantly populated in the p53-P27N simulations. In this intermediate Phe19, one of the key residues involved in binding with MDM2, is not in the helical form (Fig. 3c). We speculate that if the formation of this intermediate can be reduced (i.e. negatively designed for), the probability for the peptide to be more helical would increase. A similar intermediate is also populated in other p53 peptides, albeit to a smaller extent (less than 1%, data not highlighted in Fig. 3a). We also speculate that in addition to the reasons hypothesized in the previous study^14^, the lower affinity of P27N compared to P27S may result from the accumulation of this intermediate.

### Design of a new peptide

Our study suggests that the 12/1 peptide has a rugged energy landscape and its sequence is not optimal to fold into an α-helix. In the simulations, it populates different structures including β-sheets which are known for their aggregation propensities^39^. In order to reduce the β-sheet population and induce helicity we now propose a set of mutations in the sequence of 12/1 (MPRFMDYWEGLN).

i. Proline is known to disrupt the α-helical structure. In addition, Pro2 also affects the helical conformation of Met1 and hence it should be mutated to any other residue with a higher helical propensity.
ii. The first residue (Met1) may not be necessary: in the crystal structure 1T4F describing the complex of 12/1 with MDM2, the first residue is missing suggesting that its interactions with MDM2 may not be very stable and hence redundant.
iii. Met5 populates a left-handed helix (Fig.S6), and should be mutated to reduce the frustration in the folding of 12/1. Additionally, Met is known to interfere with intra-helical hydrogen bonding^40^ and is shown to oxidize quickly. Hence, it is not a good candidate for peptide design^41^.
iv. The residues which have equal probabilities of being in α-helical or in β-sheet conformations, as seen in our simulations, are Asp6, Tyr7 and Trp8. Large aromatic amino acids (Tyr, Trp, Phe) and β-branched amino acids have a higher propensity to be in the middle of β-sheets^42^. We propose that one or more of these residues could also be mutated so as to reduce the β-sheet population in 12/1. Since Trp8 is one of the key residues involved in binding interactions, it is best not mutated. Tyr7 could be mutated to Leu, since Leu in this position (Leu22) appears to play an important role in maintaining rigidity in the p53 peptides and has the highest helical propensity in our p53 peptide simulations. Leu also has the 3^rd^ highest helical propensity after Ala and Arg^43^. We do not mutate charged amino acids, as a previous study suggested that they may be important in the binding of the 12/1 peptide to MDM2^14^. We generated several sequences of putative mutant peptides and assessed their helical propensities using the program Agadir^44^, which successfully predicted the order of helicity of the other peptides (Table S1). We chose the sequence with the highest helical propensity (25-fold higher than for 12/1; Table S1). This sequence is ‘-**T**RF**A**D**L**WE**L**LN’ (the ‘-’ represents the deletion of Met from 12/1; the residues in bold represent the mutations Pro2Thr, Met5Ala, Tyr7Leu and Gly10Leu in 12/1). We refer to this peptide as 12/1 m.

**Figure 6:**
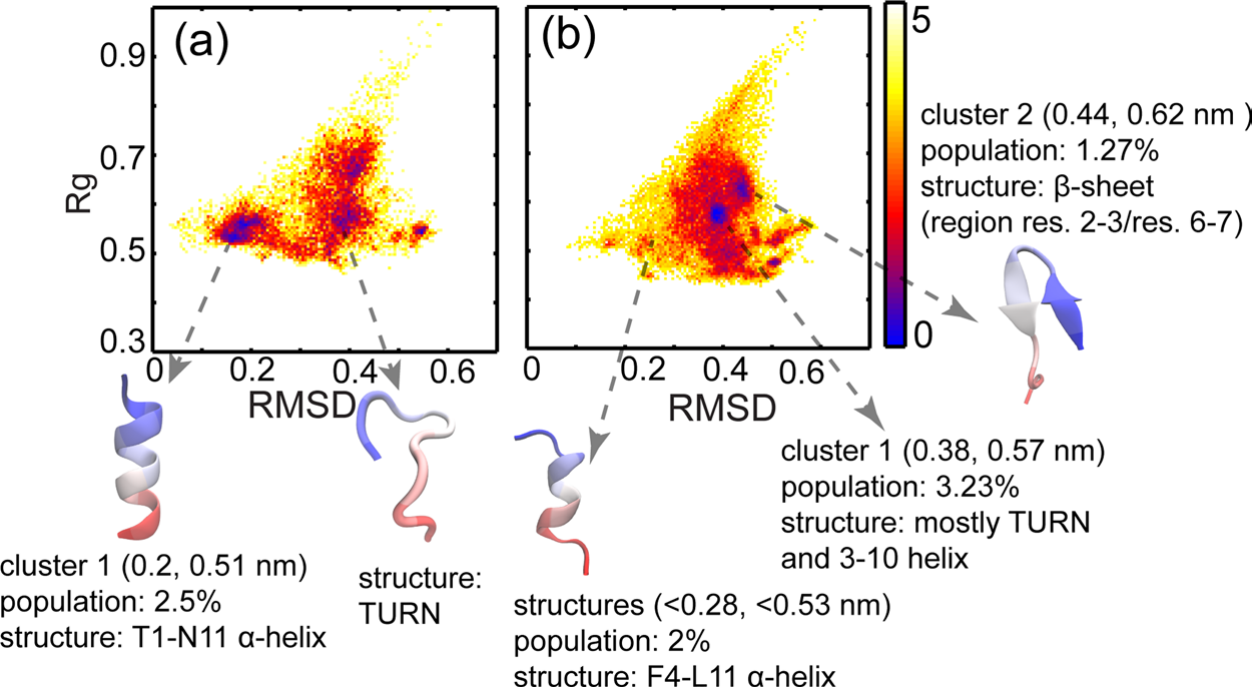
Conformational landscape of the 12/1 m peptide. The 2D free energy surfaces (2DFES) showing the distribution of the conformations of (**a**) 12/1 m, the proposed mutant peptide and (**b**) 12/1. The color scheme used is shown by the color bar on the right. The X-axes represent the root mean square deviation (RMSD; in nm) from the bound form of the p53-WT peptide (1YCR: chain-B) calculated over the Cα atoms. The Y-axes represent the radius of gyration (Rg; in nm). Representative structures from significantly populated clusters are shown, with arrows indicating their positions on the 2DFES. The structures are colored as blue to red from the N to the C-termini. Information about the clusters including the average (RMSD, Rg) coordinates of the ‘cluster centre’ are mentioned below each structure. It is clear that the 12/1 m peptide has a higher population of helical structures than 12/1.

**Figure 7:**
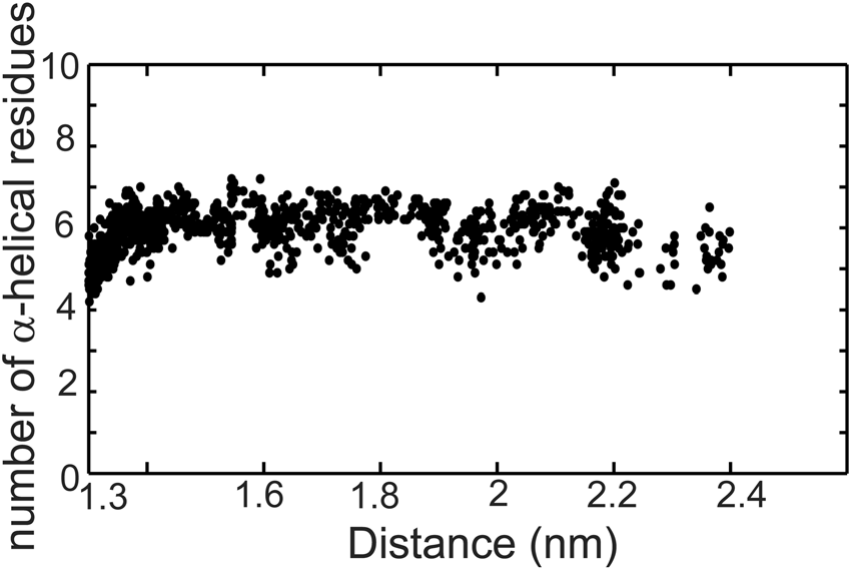
The behavior of the secondary structure of the 12/1 m peptide as it is pulled away from the MDM2 binding site. Here we show the average behavior of the peptide across ten replicates of the pulling simulations. X-axis is the distance between the centers of masses of the peptide atoms and the MDM2 atoms. Y-axis is the number of residues in α-helical conformations as calculated by DSSP. These values are calculated for each frame of a trajectory and are then averaged across all 10 trajectories for a given simulation time point. Starting distance between the centers of masses of the peptide and MDM2 is 1.33 nm. 12/1 m retains its helicity at longer distances than 12/1.

REMD simulations of 12/1 m show the percentage populations of α-helix, 3_10_-helix and β-sheet to be 23.3, 3.66 and 0 respectively (Fig. 6), suggesting a marked improvement in the helicity over the 12/1 set (Tables 2, 3). We next carried out conventional MD simulations (for 100 ns in triplicate at 300 K under NPT equilibration conditions) for the complexes of MDM2 with the WT p53 peptide, 12/1 and 12/1 m, using the PDB structure 1YCR as the template. In these simulations, the total number of contacts made by key binding residues is similar for both 12/1 and 12/1 m peptides, indicating that 12/1 m makes as many interactions with MDM2 as does 12/1 (Fig. S7). We also carried out pulling simulations of 12/1 m from MDM2 and find that the peptide remains α-helical even at distances greater than 1.8 nm (Fig. 7), at which point 12/1 had began to lose helicity (Fig. 5). These findings suggest that the 12/1 m peptide is more helical than 12/1, and its binding to MDM2 should occur predominantly through conformational selection and hence the free energy of binding is expected to be more entropically driven.

#### A comment on the pulling simulation*s*

The methodology applied in this study for pulling the peptides is limited to pulling them orthogonal to the surface of the receptor MDM2 and has been carried out in the spirit of other similar studies^24,45^; however it is to be noted that these can at best report only on the interaction stability. It is clear that this method will not exhaustively sample the different directions of approach/exit of the peptide to/from the surface of MDM2. However, it can provide some insights into the conformations adopted by the peptides at increasing distances from the receptor (as has been shown in our laboratory earlier for the disordered C-terminus of p53 binding to multiple receptors^24^), which should approximate the behaviour of the peptides in solution. From this conformational landscape, we make some inferences about the binding mechanisms. Indeed, in earlier work using Brownian Dynamics, we have seen that the channels of approach of peptides to MDM2 are indeed not necessarily those sampled by the pulling simulations employed here^46^. In a separate study aimed at understanding perplexing mass spectrometry experiments, we also showed that indeed, even small molecules like nutlin initially bind to MDM2 at a site where the C-terminus of the p53 peptides locate, and subsequently, by mechanisms that are not clear, find their crystallographically observed pose by diffusing across the surface^47^. Similar “non-orthogonal” behaviour has been reported for ligands binding to the ATP pocket in kinases^48^.

#### The role of the missing disordered region of MDM2

A question that arises is whether 1YCR, with the disordered region or the lid region (residue 1–24) missing, is a suitable enough model to study the interactions of peptides with MDM2. This flexible lid has been shown, through elegant NMR studies^49^, to adopt two major conformational states: a closed state where it occludes the binding pocket of MDM2 and an open state, where it is displaced well away from the binding pocket. The study also demonstrates that the lid is agnostic to small molecules such as nutlin, i.e. occupies both closed and open states, while when bound to peptides, it exists predominantly in the open state. Specifically, a very detailed computational study^50^ shows that the lid appears to make very little interactions with the p53-peptide. Indeed, this concurred with experimental binding data which showed that the affinity of a peptide for MDM2 representing the transactivation domain of p53 was independent of the size of the lid region in the MDM2 constructs (residue 2–118 vs 17–125); as measured by SPR and ITC^51^. Other computational studies have also highlighted the role of the lid in modulating the structural dynamics of MDM2 and of ligand binding to MDM2^52,53^. We ourselves have, in several earlier computational studies, used 1YCR to successfully rationalize the binding of the transactivation domains of p53, p63 and p73 and also to successfully design peptides that have shown potency in biophysical and biological studies^54–57^. Of course while this does not comment on the putative interactions between the lid and the peptides totally, especially at the C-terminus end of the peptides as they are located closer to the lid region and are known to have allosteric effects^1^, nevertheless it suggests that 1YCR lacking the lid, is a good model system for understanding the interactions between MDM2 and peptides.

## Electronic supplementary material

Supplementary Information
