# Supplementary material for: Characterizing the conformational landscape of MDM2-binding p53 peptides using Molecular Dynamics simulations
Source: Sci Rep. 2017 Nov 15;7:15600. doi: 10.1038/s41598-017-15930-4 (PMC5688104; doi:10.1038/s41598-017-15930-4)
Supplement: Supplementary file 1 — Supplementary Information [file 41598_2017_15930_MOESM1_ESM.pdf]

Supplementary Information for

Characterizing the conformational landscape of MDM2-binding p53 peptides using Molecular Dynamics Simulations

Shilpa Yadahalli[1,2,3,4], Jianguo Li[2,5], David P. Lane[4], Shachi Gosavi[1], Chandra S. Verma[2,4,5]

[1]Simons Centre for the Study of Living Machines, National Centre for Biological Sciences, Tata Institute of Fundamental Research, Bellary Road, Bangalore-560065, India.

[2]Biomolecular Modeling and Design Division, Bioinformatics Institute, A*STAR (Agency for Science, Technology and Research), 138671, Singapore

[3]Manipal University, Madhav Nagar, Manipal-576104, India.

[4]p53 Laboratory, A*STAR (Agency for Science, Technology and Research), 138648, Singapore

[5]Singapore Eye Research Institute, 11 Third Hospital Avenue, #06-00, Singapore 168751.

[6]Department of Biological Sciences, National University of Singapore, 16 Science Drive 4, Singapore 11758

[7]School of Biological Sciences, Nanyang Technological University, 60 Nanyang Drive, Singapore 637551.

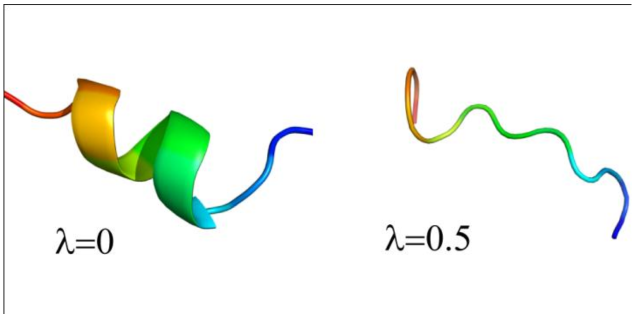

Figure S1. Representative conformations of the p53 peptide in the two extreme states. λ is the extent to which the Hamiltonian has been perturbed in the current study. To decide the λ value, we performed conventional MD simulations at λ= 0.4, 0.5, 0.7 and found that for λ>0.5, the peptide unfolded within 20 ns. Eight replicas were then chosen with equidistant λ values between 0 and 0.5 to run the Hamiltonian replica exchange simulations.

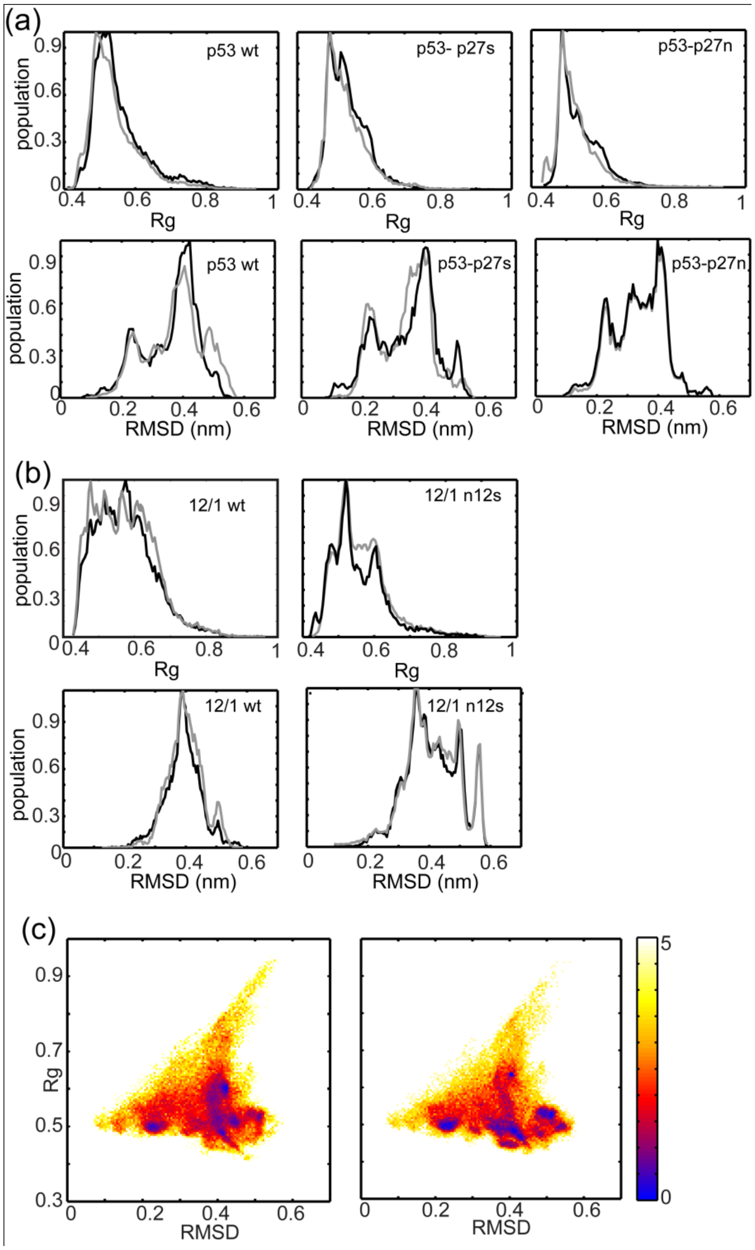

Figure S2. Testing convergence of replica exchange simulations. HREM simulations are run for a total of 300 ns for each peptide. We divide this data into 150 ns each and plot the distribution of RMSD and Rg for these two parts separately; panels (a) and (b) show the distributions for the p53 and the 12/1 peptides respectively. Black and grey represent the frames from 0-150 ns and 151-300 ns respectively. For all the peptides, black and grey show similar distributions. The RMSD is calculated for the Cα atoms

with the bound forms of the peptides as reference. (c) 2DFES is plotted for the p53-WT peptide separately for 0-150 ns and 151-300ns and again it is clear that the major clusters are similarly sampled.

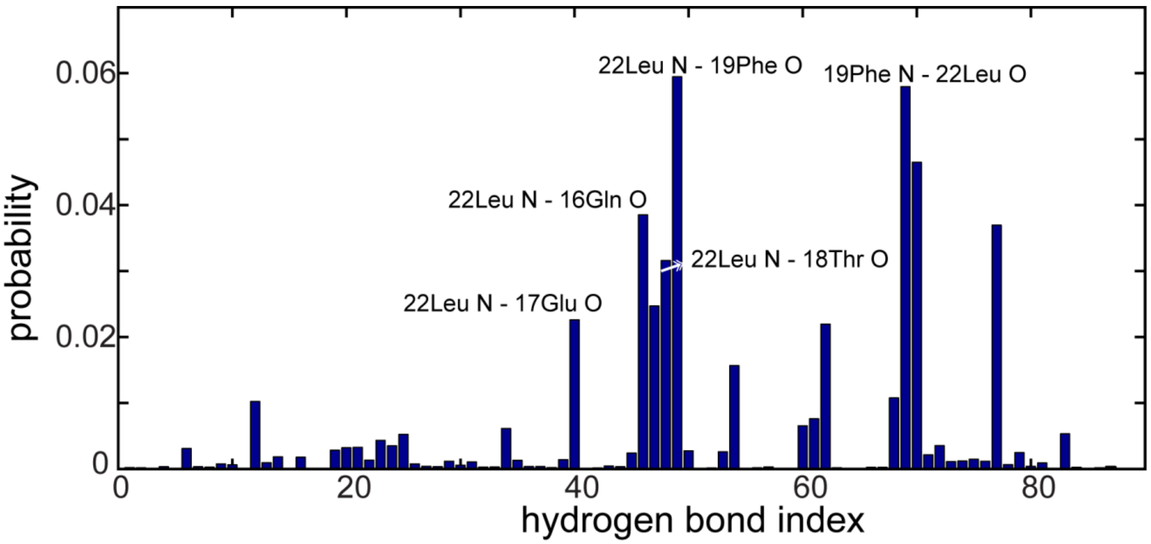

Figure S3. Probability of backbone hydrogen bond formation in the p53-P27S peptide. X-axis is the index of hydrogen bonds formed between backbone-atoms of the peptide and Y-axis is the probability of formation of a given hydrogen bond calculated from its replica exchange simulations. The hydrogen bonds formed by Leu22 are labeled on the graph. Several of the high probability hydrogen bonds have one atom from Leu22. We argue that this could be one of the reasons for the constrained (φ, ψ) angles (see Fig.4 in main text) in Leu22.

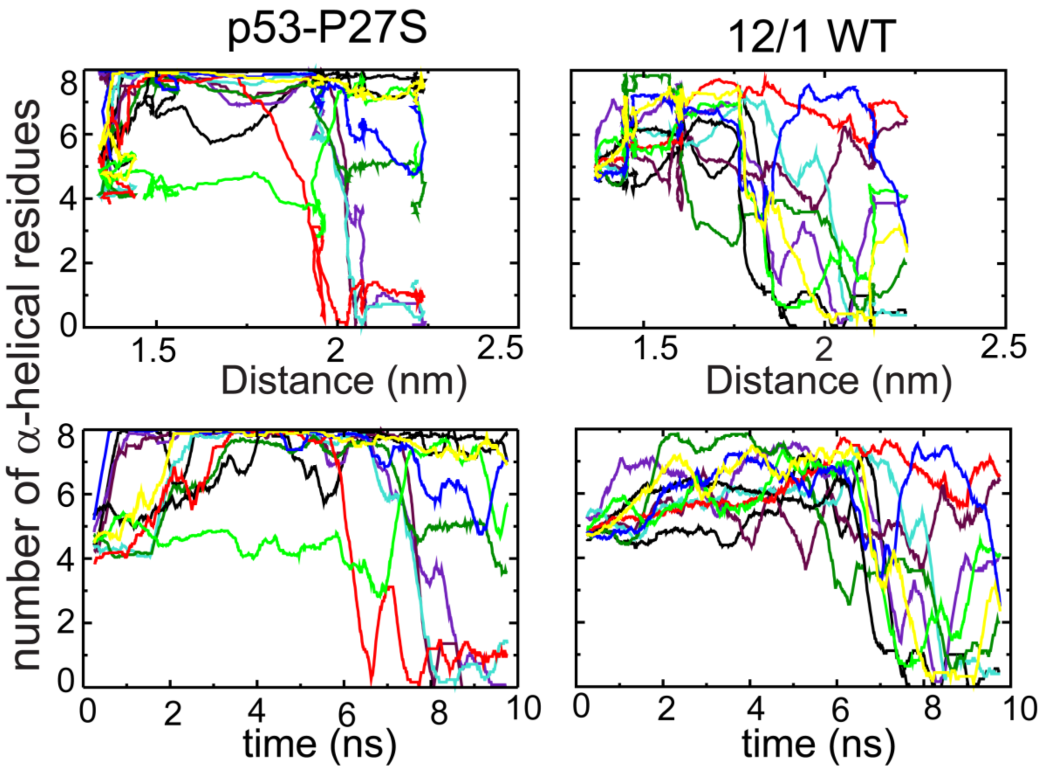

Figure S4. The behavior of the secondary structures as the peptides are pulled away from the MDM2 binding site. Number of residues adopting α-helical conformations as given by DSSP are plotted against the distance between the center of masses of the peptide and MDM2 atoms (upper row) and the simulation time (lower row). Different colors represent different replicates. There are a total of 10 replicates. The plots on the left side correspond to p53-P27S and those on the right side are for the 12/1 WT peptide. p53-P27S is more helical with 7 out of 10 replicates retaining their helical conformations. The 12/1 peptides retain their helicity only until ~6 ns in 8 out of 10 replicates by which time the peptides are at a distance of ~1.75 nm from the binding site. For ease of visualization, the smoothened plots are shown.

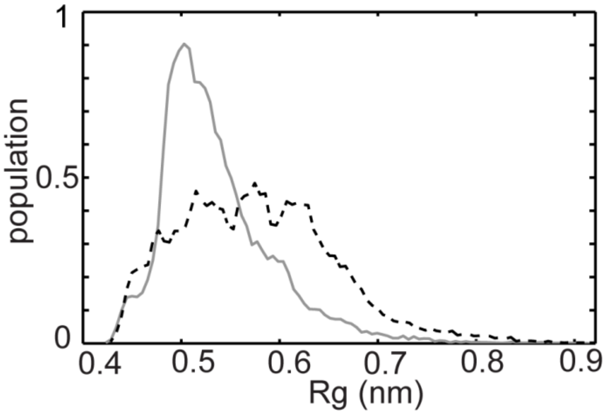

Figure S5. Distributions of the radius of gyration Rg (in nm) for p53-P27S (grey solid line) and 12/1 WT (black dotted line) peptide are shown. X axis is Rg in nm and Y axis is the normalized population from the replica exchange simulations of the peptide. The p53-P27S peptide has a higher population of conformations with Rg<0.55 nm. The broader distribution of the 12/1 WT peptide implies that it is more flexible/extended.

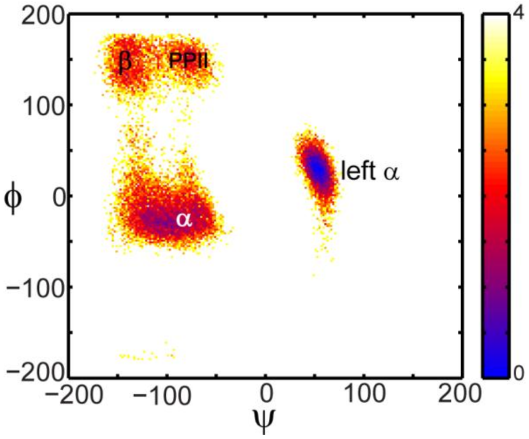

Figure S6. Ramachandran plot of Met5 from the 12/1 WT peptide. The distribution of the (-ln(population)) from replica exchange simulations of the Met5 residue from 12/1 WT peptide plotted as a function of the φ (X-axis; in degrees) and ψ (Y-axis; in degrees) angles. The distribution is colored from blue (highest density of structures) through red to yellow (lowest density of structures) with the color scheme shown by the colorbar on the right. α-helix has ((φ, ψ) ~(-60°, -45°)), β-sheet has ((φ, ψ) ~(-135°, 135°)), PPII has ((φ, ψ) ~(-75°, 150°)) and left-handed α-helix has ((φ, ψ) ~(60°, 45°)). Met-5 populates significant amounts of left handed α-helix.

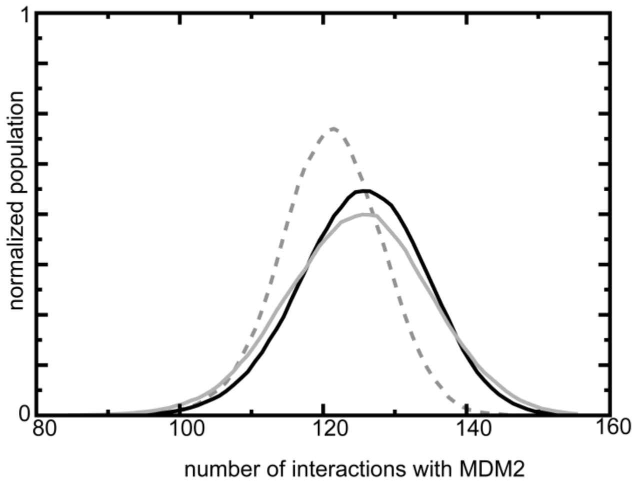

Figure S7. Analysis of the binding of 12/1 peptides to MDM2. In order to lend support to the premise that the 12/1m peptide can bind to MDM2 we carry out conventional MD simulations of the MDM2-peptide complexes. The number of interactions MDM2 makes with the 3 key binding residues of the peptide (Phe19, Trp23 and Leu26) was calculated for the last 50 ns out of a total 100 ns trajectory. A 4.0 Å cutoff at the all atom level was used to define an interaction. The curve in the figure shows the distribution of the total number of such interactions in the 12/1 (grey), the 12/1m (black) and p53-WT peptide (black) simulations. The X-axis gives the total number of contacts per snapshot and the Y-axis is the normalized population of snapshots which have a given number of contacts. The two population distributions are similar; indicating that on an average the 12/1m makes more interactions with MDM2 than p53-WT and as many interactions as 12/1.

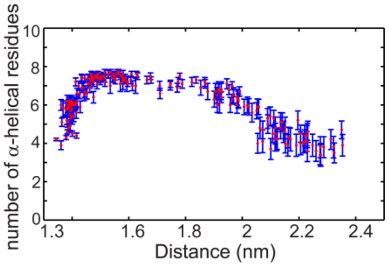

(a)

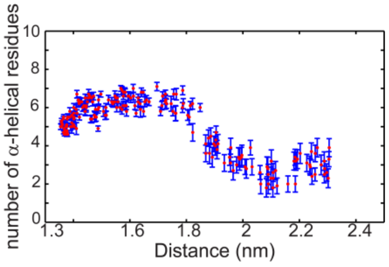

(b)

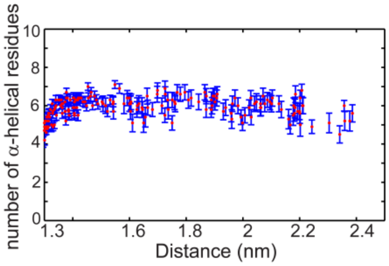

(c)

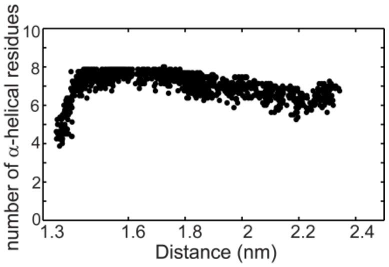

(d)

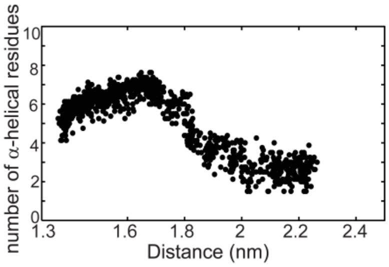

(e)

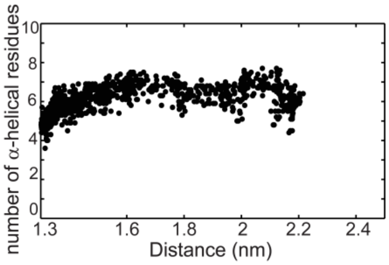

(f)

Fig. S8. Testing the convergence of pulling simulations. We have carried out error estimates, using the jackknife method[25], on the pulling simulations of: (a) p53-P27s (b) 12/1 (c) 12/1m. The size of the error bars shown here is twice the square root of the variance and the variance was calculated using the jackknife method by re-sampling to 9/10ths of the total simulation dataset. We have plotted only every 5th data point for ease of visualization; but the entire dataset was used for the error estimation. Data points are shown by red dots and the error bars are shown in blue lines. We have also carried out another set of 10 replicates of pulling simulations on these three systems and the results are shown in the lower panel: (d) p53-P27s (e) 12/1 (f) 12/1m. It is clear that the trends are the same in the two sets of simulations.

Peptide sequence	Name	Percentage helicity from AGADIR
Q_{16}ETFSDLWKLL**P_{27}**	P53-wild type (WT)	0.79
QETFSDLWKLL**S**<br>QETFSDYWKLL**S**	p53-P27S	1.04<br>0.47
QETFSDLWKLL**N**	p53-P27N	1.74
MPRFMDYWEGL**N**<br>MPRFMD**L**WEGLN<br>-**T**RFMD**L**WEGLN<br>-**T**RF**A**D**L**WEGLN<br>-**T**RF**A**D**L**WE**L**LN<br>-**P**RF**A**D**L**WE**L**LN<br>-**E**RF**A**D**L**WE**L**LN	12/1WT	0.11<br>0.13<br>0.71<br>1.05<br>2.62<br>0.75<br>0.88
MPRFMDYWEGL**S**	12/1-N12S	0.1

Table S1: Percentage helicity values calculated using AGADIR are shown. The amino acids in red correspond to the mutations.
